# Birth preparedness as a precursor to reduce maternal morbidity and mortality among pregnant mothers in Medebay Zana District, Northern Ethiopia

**DOI:** 10.1186/s13104-019-4331-z

**Published:** 2019-05-28

**Authors:** Hailay Gebreyesus, Tesfay Berhe, Mebrahtu Teweldemedhin

**Affiliations:** 1 Department of Public Health, College of Health Science, Aksum University, P.O. Box 298, Aksum, Ethiopia; 2 Department of Medical Laboratory Science, College of Health Sciences, Aksum University, Aksum, Ethiopia

**Keywords:** Birth preparedness, Pregnant women, North Eastern, Ethiopia

## Abstract

**Objectives:**

Most maternal and newborn deaths occur during labour and delivery. Birth preparedness and complication readiness practice getting early services when problems may arise is the most achievable components of safe motherhood strategies. However, there is limited evidence found particularly in the study area. Thus, a community based cross-sectional study was conducted from May 17 to June 30, 2017, to assess birth preparedness practice and associated factors among pregnant women in Medebay Zana district in Northern Ethiopia.

**Result:**

The finding showed that about 176 (32%) of the respondents were prepared for birth based on the criteria set in this study. Preparation for birth was higher among married women (AOR = 4.14, 95% CI (1.47–11.64)), among governmental employed women (AOR = 2.69, 95% CI (1.19–6.05)), those who attend antenatal care service (AOR, 0.11, 95% CI (0.05–0.22)), planned pregnancy (AOR = 0.06, 95% CI (0.03–0.15), those who had saving habit (AOR = 15.81, 95% CI (7.20–34.72), duration of pregnancy near to 9 month (AOR = 5.86, 95% CI (3.25–10.58). Preparation for birth was lower among illiterate mothers (AOR = 0.15, 95% CI (0.07–0.30), among mothers who attended primary education (AOR = 0.01, 95% CI (0.01–0.04)). The prevalence of birth preparedness practice in the study area was low. Community-based health education about preparation for birth is important.

**Electronic supplementary material:**

The online version of this article (10.1186/s13104-019-4331-z) contains supplementary material, which is available to authorized users.

## Introduction

Most of the time pregnancy and childbirth are times of joy for parents, families, and communities at large [1, 2]. But in many countries where healthcare service coverage and quality remain low, they could also be periods of great risk to the health and survival of women and newborns [3, 4].

Globally, around 1500 women die every day from complications related to pregnancy and childbirth [1, 5–7]. Developing countries account for 99% of the global maternal deaths and the great share (62%) belongs to the sub-Saharan Africa region. Almost 80% of maternal morbidity and mortality worldwide are directly related to pregnancy and childbirth complications [8]. Obstetric related complications including severe hemorrhage, infection, hypertensive disorders, sepsis and obstructed labor, and unsafe abortion are among the key factors to maternal death [8]. An estimated 125,000 women and 870,000 newborns die every year in Africa, particularly in the 1st week after birth [9]. Ethiopia is one of six countries sharing 50% of the total global burden of maternal mortality [10]. One of the reasons is the lack of birth preparedness and complication readiness, which is recognized as the most cost-effective and achievable components of safe motherhood programs around the world [11, 12].

Birth preparedness and complication are an inclusive strategy aimed to promote timely use or access to skilled maternal and neonatal health services during childbirth or obstetric emergencies. It reduces any types of delay through early preparation and decision-making, identifying a skilled birth attendant, support in the family, saving money ad means of transportation, identifying health facility and identification of compatible blood donors when needed [12, 13]. Preparedness and complication have been associated with reduced maternal and newborn morbidity and mortality through increased preventive behaviors of mothers and families [14–21].

Even though, the government of Ethiopia has introduced and is currently implementing a policy that provides free maternal healthcare services to all pregnant women and new babies in all governmental healthcare facilities but different studies conducted in Ethiopia showed that birth preparedness and complication readiness among pregnant women was still very low (22% and 17%) [19, 22]. However, there are no studies which documented birth preparedness and complication readiness practice among pregnant women in the study area. Thus, the aimed of this was to assess the magnitude and associated factors of birth preparedness practice among pregnant women.

## Main text

### Methods

#### Study design and setting

A community-based cross-sectional study was carried out from May 17 to June 30, 2017, in Medebay Zana district. Medebay Zana is one of the administrative districts of North West Zone of Tigray region, located about 1057 km away from Addis Ababa. It has 20 smallest Administrative Unit, 7 health centers, and 22 health posts. According to the district health office report in January 2017, the district has an estimated population of 147,822. The proportion of pregnant women constitutes 3.4% of the total population that is 5026.

All pregnant women within the district and all sampled pregnant women within the selected households of the kebeles were the sources and the study population respectively. All self-reporting pregnant mothers who lived in the selected Kebele for at least 6 months were included and those who are unable to give appropriate responses due to their physical and mental illness were excluded from the study.

#### Sample size and sampling techniques

The sample size was determined by using a single population proportion formula using the following assumption: Proportion of pregnant women who were prepared for birth/have birth plan (p) 17% of birth preparedness practice among pregnant women in southern Ethiopia [11], margin of error (d) = 3% and 95% confidence level and 5% for possible non response rate. So the final sample size was 552.

From the 20 kebeles of the district, 6 were randomly selected; the sample was taken from selected kebeles proportional to population size and then a simple random sampling technique was used to select study participants within each kebele. List of pregnant mothers with their household numbers was taken from community health information system registration prepared by health extension workers as the sampling frame then independent sampling frame was developed for each kebele. The data collection was continuous until the predetermined sample size obtained.

#### Operational definitions

##### Birth preparedness practice

The pregnant woman was asked whether she followed the desired elements of birth preparedness practice. Which consists of 4 items, the responses were scored as “0” for no answer and “1” for yes answer. And the total score was obtained by computing all items, which range from 0 to 4 points. Respondents scores ≥ 3 was considered as being well-prepared and the rest as not well prepared for birth.

If pregnant mother she can mention at least three of the key four danger signs during labour/childbirth (severe vaginal bleeding, prolonged labour (> 12 h), convulsion and retained placenta) spontaneously, she was considered as knowledgeable otherwise not knowledgeable.

If she can mention at least two of the three key danger signs during pregnancy (vaginal bleeding, swollen hands/face, and blurred vision) spontaneously, she was considered as knowledgeable otherwise not knowledgeable [22, 23].

##### Data collection tool

The data collection tool was adopted from the safe motherhood questionnaire developed by maternal and neonatal health program of JHPIEGO the affiliate of Johns Hopkins University according to the context and objectives of the study [12]. The questionnaire was prepared in English and translated to local language (Tigrigna) and translated back to English by another translator. Five % of the questionnaire was pre-tested in a similar setting; correction and modification were done based on the gap identified during the interview. The questionnaire was grouped into four categories; Socio-demographic characteristics; health service utilization factors; obstetric characteristic and Knowledge on danger signs of pregnancy, labor, and delivery.

#### Data collection technique and quality control

Data were collected by four clinical nurses using a pre-tested structured questionnaire and supervised by two general public health experts and the principal investigator. Training was given for 2 consecutive days on the aim of the research, content of the questionnaire and how to conduct the interview for data collectors and supervisors to increase their performance in field activities. Strict supervision was done by supervisors and principal investigators.

##### Data processing and analysis

The data were checked and edited, then coded, entered into EPI Info version 7.1.2 and exported to SPSS Version 21 for analysis, cleaned and checked for outliers and completeness. Frequency, percentages, proportions, odds ratios, and logistic regressions were computed. Variables with a P < 0.05 and AORs with the 95% confidence interval were considered as significant.

### Result

#### Socio-demographic characteristics

In this study, a total of 552 pregnant women were interviewed. All eligible pregnant women selected in the samples responded to the questionnaire. The mean age of the participants was 26.9 (SD of 6.9) years. Majority of the pregnant women 488 (88.4%) were married and 121 (21.9) pregnant women were unable to read and write (Table 1).

**Table 1:** Socio-economic and demographic characteristics among pregnant women in Medebay Zana district, July 2017

Variables	Category	N (%)
Age	≤ 20	118 (21.4)
	21–25	136 (24.6)
	26–30	154 (27.9)
	31–35	48 (8.7)
	> 35	96 (17.4)
Religion	Orthodox	523 (94.7)
	Muslim	29 (5.3)
Ethnicity	Tigrean	532 (96.4)
	Others^a^	20 (3.6)
Marital status	Married	488 (88.4)
	Not married	64 (11.6)
Educational level	Illiterate	302 (54.7.)
	Primary education	137 (24.8)
	Secondary and above education	113 (20.5)
Occupation	Housewife	347 (62.9)
	Governmental employed	77 (13.9)
	Merchant	73 (13.2)
	Others^b^	55 (10.0)
Monthly family income	< 650 Birr	208 (37.7)
	651–1500 Birr	182 (33.0)
	> 1500 Birr	162 (29.3)
Family size	0–2	420 (76.1)
	3–4	90 (16.3)
	≥ 5	42 (7.6)

^a^Amhara, Afar and Yem

^b^Daily laborers and privet employed

#### Antenatal care visits during the current pregnancy

About 450 (81.5%) planned their pregnancy and 376 (68.1%) attended ANC checkup; only 218 (58%) of the pregnant women attended ANC 4 or more times during their current pregnancy. Half of them 276 (84.4%) got advice on birth preparation during their ANC visit (Additional file 1: Table S1).

#### Obstetric characteristics of the respondents related to their previous pregnancy

Two hundred seventy-one (49.1%) of the respondents were pregnant for the first time. Majority of them (79.4%) were gravid 1–2 and five (1.8%) of the pregnant women had a history of stillbirth (Additional file 2: Table S2).

#### Birth preparedness practices

Out of the 552 pregnant women, 176 (32%) were considered as being well prepared to give birth. Three hundred thirty-five (60.7%) respondents reported that they saved money, 179 (32.4%) identified means of transportation, 371 (67.2%) identified their place of delivery and 173 (31.3%) mothers had already identified skilled birth attendant (Table 2).

**Table 2:** Practices of respondents on preparation for birth with current pregnancy among pregnant women in Medebay Zana district, July 2017

Variables	Category	N (%)
Saved habit	Yes	335 (60.7)
	No	217 (39.3)
Arranged transport	Yes	179 (32.4)
	No	373 (67.6)
Identified a skilled birth attendant	Yes	173 (31.3)
	No	379 (68.7)
Identified facility for delivery	Yes	371 (67.2)
	No	181 (32.8)
Place of delivery	Health institution	293 (79)
	Other place^a^	78 (21)
Final decision maker on birth place	Both	261 (47.3)
	Women	211 (38.2)
	Husband	80 (14.5)

^a^Home or out any health institutions

#### Factors associated with birth preparedness practices

In the multiple variable logistic regression analysis, marital status, educational status, planned pregnancy, ANC care, saving habit and duration of pregnancy remained significantly associated with birth preparedness practice. Those women who were in marital union were 4.14 times more likely to be prepared for birth than those who were not in marital union (AOR = 4.14, 95% CI (1.47–11.64)). Those illiterate pregnant women were 6.67 times less likely to be prepared for birth than those who were attended secondary and above education (AOR = 0.15, 95% CI (0.07–0.30)) and pregnant women who attended primary education were 100 times less likely to be prepared for birth than those who were attended secondary and above education (AOR = 0.01, 95% CI (0.01–0.04)). Mothers who were not planned to their pregnancy were 16.67 times less likely to practice birth preparedness when compared to those who had planned pregnancy (AOR = 0.06, 95% CI (0.03–0.15)). Mothers who had saving habit for their labor and delivery were 15.81 times more likely to be prepared for birth (AOR, 15.81, 95% CI (7.20–34.72)). Pregnant women who were attended ANC follow up for their pregnancy were 9.09 times more likely to be prepared for birth (AOR, 9.09, 95% CI (0.05–0.22)). Pregnant women who were their pregnancy duration 7–9 months were 5.86 times more likely to practice birth preparedness as compared to those who were their pregnancy duration 3–6 months (AOR = 5.86, 95% CI (3.25–10.58)) (Table 3).

**Table 3:** Factors associated with birth preparedness practices among pregnant women in Medebay Zana district, July 2017

Variables	Birth preparedness practice		COR (95% CI)	AOR (95% CI)
	No n (%)	Yes n (%)		
Marital status				
In marital union	321 (65.8)	167 (34.2)	3.12 (1.53–6.60)*	4.14 (1.47–11.64)*
Not in marital union	55 (85.9)	9 (14.1)	1	1
Educational status				
Illiterate	217 (71.6)	86 (28.4)	0.16 (0.10–0.25)*	0.15 (0.07–0.30)*
Primary	127 (93.4)	9 (6.6)	0.03 (0.01–0.06)*	0.01 (0.01–0.04)*
Secondary and above	32 (28.3)	81 (71.7)	1	1
Occupation				
House wife	285 (82.1)	62 (17.9)	1	1
Governmental employed	39 (50.6)	38 (49.4)	4.46 (2.47–7.57)*	2.69 (1.19–6.05)*
Merchant	27 (37.0)	46 (63.0)	7.83 (4.52–13.56)*	4.02 (1.73–9.37)*
Others	25 (45.5)	30 (54.5)	5.52 (3.04–10.03)	5.27 (2.17–12.80)*
Planned pregnancy				
Yes	250 (66.5)	126 (33.5)	1	1
No	126 (71.6)	50 (28.4)	1.31 (0.86–1.88)	0.06 (0.03–0.15)*
ANC care				
Yes	181 (58.8)	127 (41.2)	2.79 (1.89–4.11)*	0.11 (0.05–0.22)*
No	195 (79.9)	49 (20.1)	1	1
Saving habit				
Yes	101 (46.5)	116 (53.5)	5.26 (3.56–7.75	15.81 (7.20–34.72)*
No	275 (82.1)	60 (17.9)	1	1
Arranged transport				
Yes	127 (70.9)	52 (29.1)	1.22 (.82–1.79)	1
No	249 (66.8)	124 (33.2)	1	
First pregnancy				
Yes	220 (81.2)	51 (18.8)	1	1
No	156 (55.5)	125 (44.5)	3.46 (2.35–5.08)*	1.22 (0.63–2.35)
Duration of pregnancy (months)				
3–6	101 (51.0)	97 (49.0)	3.34 (2.30–4.86)	1
7–9	275 (77.7)	79 (22.30)	1	5.86 (3.25–10.58)*
Knows danger signs of labor and delivery				
Yes	197 (91.6)	6 (4.6)	1	
No	124 (95.4)	18 (8.4)	1.89 (0.73–4.89)	0.76 (0.15–3.96)

*ANC* antenatal care, *AOR* adjusted odds ratio, *CI* confidence interval, *COR* crude odds ratio

1: referent

* Significant at p value < 0.05

### Discussion

This community based cross-sectional study assessed the magnitude and factors associated with birth preparedness. The proportion of birth preparedness practice was found to be 32%. This finding is higher than the figures of other study conducted in Ethiopia [11, 22] but lower than that of Nigeria [24] and India [17]. This could be due to the socio-economic status of the participants and or access to health facility.

In this study, marital status, educational status, planned pregnancy, ANC care, saving habit, and duration of pregnancy were significantly associated with birth preparedness. Married women were more likely to be prepared for birth than those who were not married. This could be because; married women may have supported by their husbands to prepared and utilized maternal healthcare. Similar findings have been observed in the studies conducted in Tanzania, Uganda, North Ethiopia and Indore city of India [17, 20, 22, 25]. This might be due to husband’s knowledge and awareness towards birth preparedness.

Mothers’ occupational status was significantly associated with birth preparedness in this study. Those pregnant women who were governmental employed, merchants and others (daily laborers and privet employed) in their occupational status were more likely to be prepared for birth than those who were housewife. Similar finding was found in a study done in Ethiopia and India [17, 22]. Pregnant women with planned pregnancy were more likely to be prepared for birth than those with an unintended pregnancy. Those mothers with planned pregnancy already accepted it and try their best to get prepared for birth. Those pregnant women who attended ANC were found to be more likely to be prepared for birth. ANC is important to offer advice and information about birth preparedness, danger signs of obstetric complications and emergency preparedness. Birth preparedness is a basic element of ANC aimed to reduce and unnecessary delays and hence improves maternal and fetal outcomes [12]. Additionally, any visit to a health facility was significantly associated with birth preparedness in some other studies [11, 20, 22, 26]. The current study also identified pregnant women who had saving habit during their pregnancy. The results showed that about 53.5% of the respondents had saving habit during their current pregnancy by saving money, identifying a means of transportation and identifying skilled personnel. Transportation is a barrier to reach the health facilities and seek care; it could be difficult to secure transport after labor has already occurred. Saving money and arranging transport ahead of time reduces the delay in reaching the appropriate place where service is given. In this study, more than half of the respondents had saving habit and have identified a means of transportation ahead of time which is higher as compared to a study conducted in Ethiopia [11]. In this study duration of pregnancy was significantly associated with birth preparedness. Those mothers who were clothed their pregnancy duration to 9 month was more likely to be prepared for birth as compared to those who were not clothed their pregnancy duration 9 month (AOR = 5.86, 95% CI (3.25–10.58). this finding is similar with previous studies in Ethiopia [11, 16].

In this study 1.8% of the pregnant women had a stillbirth which is lower as compared to previous studies conducted in Ethiopia which was 9.3 and 5.9% respectively [16, 27]. In general, the magnitude of birth preparedness practice is low in the study area. Marital status, educational status, planned pregnancy, ANC follow up, saving habit and duration of pregnancy were factors associated with birth preparedness practice. Thus, sustainable and comprehensive community-based education about preparation for birth and empowerment of women by expanding educational opportunities are important factors in enhancing birth preparedness.

## Limitation of the study

As it was a cross-sectional study, causality may not be established and since the participants have not completed their period of pregnancy, they may have the opportunity or need to make arrangements related to birth preparedness practice than the currently reported prevalence may underestimate the real prevalence on the ground.

## Additional files

**Additional file 1: Table S1.** Antenatal care services and awareness on obstetric danger signs among pregnant women in Medebay Zana district, July 2017.

**Additional file 2: Table S2.** Obstetric characteristics related to the previous pregnancy among pregnant women in Medebay Zana district, July 2017.

## Data Availability

The datasets in which conclusion taken is available in the form of Microsoft Excel. It is available on requesting.

## Abbreviations

ANC: antenatal care
BP: birth preparedness
DHS: demographic and health survey
HEW: health extension worker
TBA: traditional birth attendants
TTBA: trained traditional birth attendants
SPSS: Statistical Package for Social Science
USA: United State of America
WHO: World Health Organization
