# Supplementary material for: Birth preparedness as a precursor to reduce maternal morbidity and mortality among pregnant mothers in Medebay Zana District, Northern Ethiopia
Source: BMC Res Notes. 2019 May 28;12:304. doi: 10.1186/s13104-019-4331-z (PMC6540634; doi:10.1186/s13104-019-4331-z)
Supplement: Supplementary file 1 — Additional file 1: Table S1. Antenatal care services and awareness on obstetric danger signs among pregnant women in Medebay Zana district, July 2017. [file 13104_2019_4331_MOESM1_ESM.docx]

| Variable | Category | N (%) |
| --- | --- | --- |
| Planned pregnancy | Yes | 450(81.5) |
|  | No | 102(18.5) |
| ANC checkup | Yes | 376(68.1) |
|  | No | 176(31.9) |
| Number of ANC visit (N=376) | 1 | 347(92.3) |
|  | 2 | 328(87.2) |
|  | 3 | 322(85.6) |
|  | >/=4 | 218(58 ) |
| Training of first ANC | 3-6 months | 368(97.8) |
|  | 7-9 months | 8(2.2) |
| Personnel Checked | Health professionals | 361(96.0) |
|  | Others * | 13(4.0) |
| Advice given during ANC visit | Yes | 325(86.4) |
|  | No | 51(13.6) |
| Do you know danger signs | Yes | 332(60.1) |
|  | No | 220(39.9) |
| Knowledge on danger signs of pregnancy | Knowledgeable | 206(62.0) |
|  | Not knowledgeable | 126(38.0) |
| Knowledge on danger signs of labour and child birth | Knowledgeable | 256(77.2) |
|  | Not knowledgeable | 76(22.8) |

**Note: ANC:** Antenatal care**;** *TBA, TTBA, Husband, no one checked
