# Supplementary material for: Birth preparedness as a precursor to reduce maternal morbidity and mortality among pregnant mothers in Medebay Zana District, Northern Ethiopia
Source: BMC Res Notes. 2019 May 28;12:304. doi: 10.1186/s13104-019-4331-z (PMC6540634; doi:10.1186/s13104-019-4331-z)
Supplement: Supplementary file 2 — Additional file 2: Table S2. Obstetric characteristics related to the previous pregnancy among pregnant women in Medebay Zana district, July 2017. [file 13104_2019_4331_MOESM2_ESM.docx]

| Variables | Category | N (%) |
| --- | --- | --- |
| Duration of pregnancy during data collection | 3-6 months | 198(35.9) |
|  | 7-9 months | 354(64.1) |
| First pregnancy? | Yes | 271(49.1) |
|  | No | 281(50.1) |
| Gravida (N=281) | 1-2 | 224(79.7) |
|  | >=3 | 57(20.3) |
| Parity | 1 | 19(6.8) |
|  | 2-4 | 223(79.4) |
|  | >=5 | 39(13.9) |
| History of abortion | Yes | 6(2.1) |
|  | No | 275(97.9) |
| History of still birth | Yes | 5 (1.8) |
|  | No | 276(98.2) |
| History of infant death | Yes | 9(3.2) |
|  | No | 262(96.8) |
